# LncRNA MIR503HG inhibits cell migration and invasion via miR‐103/OLFM4 axis in triple negative breast cancer

**DOI:** 10.1111/jcmm.14344

**Published:** 2019-05-06

**Authors:** Jia Fu, Guanjun Dong, Hui Shi, Junfeng Zhang, Zhaochen Ning, Xingna Bao, Chenjie Liu, Jing Hu, Minghui Liu, Bin Xiong

**Affiliations:** ^1^ Academy of Basic Medicine Jining Medical University Jining China; ^2^ Clinical Medical School Jining Medical University Jining China

**Keywords:** biomarker, breast cancer, LncRNA, metastasis, miR‐103, MIR503HG, mircroRNA, OLFM4

## Abstract

Long non‐coding RNA MIR503 host gene (MIR503HG) is located on chromosome Xq26.3, and has been found to be deregulated in many types of human malignancy and function as tumour suppressor or promoter based on cancer types. The role of MIR503HG in breast cancer was still unknown. In our study, we found MIR503HG expression was significantly decreased in triple‐negative breast cancer tissues and cell lines. Furthermore, we observed low MIR503HG expression was correlated with late clinical stage, lymph node metastasis and distant metastasis. In the survival analysis, we observed that triple‐negative breast cancer patients with low MIR503HG expression had a statistically significant worse prognosis compared with those with high MIR503HG expression, and low MIR503HG expression was a poor independent prognostic factor for overall survival in triple‐negative breast cancer patients. The study in vitro suggested MIR503HG inhibits cell migration and invasion via miR‐103/OLFM4 axis in triple negative breast cancer. In conclusion, MIR503HG functions as a tumour suppressive long non‐coding RNA in triple negative breast cancer.

## 1 INTRODUCTION

As the most common types of cancer among women in the world with an estimated 2,088,849 new cases at 2018, breast cancer is responsible for 15.0% of deaths in females.^1^ At present, breast cancer has been recognized as a heterogeneous disease which can be categorized into different pathological subtypes according to the status of estrogen receptor (ER) and progesterone receptor (PR) and human epidermal growth factor receptor 2 (HER2).^2^, ^3^ Triple negative breast cancer is the most aggressive subtype accounting for 10%–20% of all breast cancer cases, and characterized by lacking expression of ER, PR and HER2.^4^, ^5^ Due to the lack of effective treatment including target therapy and endocrine therapy, it is useful to elucidate the mechanisms about triple negative breast cancer progression for developing novel therapies.

In our previous study, we identified olfactomedin 4 (OLFM4) as tumour‐suppressing gene to suppress cell migration and invasion through controlling MMP9 in triple negative breast cancer.^6^ Meanwhile, down‐regulation of OLFM4 expression was correlated with present lymph node metastasis, distant metastasis, advanced clinical stage and poor prognosis in triple‐negative breast cancer patients.^6^ Furthermore, we found miR‐103 functioned as oncogenic microRNA to modulate triple negative breast cancer cell migration and invasion through targeting OLFM4 expression.^7^ Long non‐coding RNAs are a subgroup of non‐coding RNAs composed of more than 200 nucleotides.^8^, ^9^ Recently, lncRNA MIR503 host gene (MIR503HG) has been suggested to be dysregulated and function as tumour‐suppressing lncRNA in a variety of human cancers.^10^ Interestingly, we analysed the online prediction tool (Starbase v2.0; http://starbase.sysu.edu.cn/) to search for lncRNAs paired with miR‐103, and found MIR503HG has potential binding sites for miR‐103. In addition, we further observed there was a negative correlation between MIR503HG expression and miR‐103 expression in triple negative breast cancer. Therefore, we had a hypothesis that MIR503HG inhibits cell migration and invasion via miR‐103/OLFM4 axis in triple negative breast cancer. So, the aim of this research is to investigate the role of MIR503HG in regulating triple negative breast cancer cell migration and invasion.

## 2 MATERIALS AND METHODS

### 2.1 Clinical samples

Ninety‐four primary breast cancer tissue samples and 30 corresponding adjacent normal mammary tissue samples were obtained from Affiliated Hospital of Jining Medical University. All triple‐negative breast cancer tissue samples were histologically classified and diagnosed by two clinical pathologists. None of the patients have undergone anti‐tumour treatments before diagnosis. The relevant experiments was approved by Jining Medical University, and complied with the principles of the Helsinki Declaration. All patients in this study signed informed consent.

### 2.2 Cell lines

Two human triple negative breast cancer cell lines (MDA‐MB‐231 and TB549) and a human normal breast epithelial cell line (MCF‐10A) were cultured according to our previous description.^6^

### 2.3 RT‐PCR analysis

RNA isolation and MIR503HG expression determination was carried out according to previous description.^6^ The primers are as follows: MIR503HG, (forward) 5'‐AAGGAATCCTCTCCCACCATTT‐3' and (reverse) 5'‐ACTCATTTGGCGGGAAAAC‐3'. The miR‐103 expression measure was performed as described in a previous report.^7^

### 2.4 Western blot

Western blotting was performed with a SDS‐PAGE Electrophoresis System according to previous report 6 with the following antibodies: OLFM4 (Billerica, MA), MMP 9 (Cell Signalling Technology, Beverly, MA) and *β*‐actin antibody (CWBIO, Jiangsu, China).

### 2.5 Cell transfection

The full‐length sequence MIR503HG cDNA was amplified by PCR, and cloned into pcDNA3.1 express vector to make a MIR503HG overexpression vector (pcDNA‐MIR503HG). The siRNA for down‐regulating MIR503HG expression (siRNA‐MIR503HG) and non‐targeting siRNA (siRNA‐NC) were obtained from GenePharma. siRNA and plasmid transfections were conducted by using Lipofectamine 3000 (Invitrogen, Carlsbad, CA) according to the manufacturer's instructions. The miR‐103 inhibitor and miR‐103 mimics transfections were conducted in accordance with previous description.^7^

### 2.6 Bioinformatics analysis and Luciferase reporter assay

The binding sites of lncRNA and miRNA were predicted on starBase (http://starbase.sysu.edu.cn/). The MIR503HG wild‐type or mutant binding miR‐103 was inserted into the pmiR‐RB‐REPORT luciferase reporter plasmid, and named as MIR503HG‐wt and MIR503HG‐mut, respectively. MIR503HG‐wt or MIR503HG‐mut was co‐transfected with miR‐103 inhibitor or miR‐103 mimics in triple‐negative breast cancer cells with Lipofectamine 3000 (Invitrogen, Carlsbad, CA) according to the manufacturer's instructions. The determination of luciferase activity was performed according to previous report.^7^

### 2.7 Cell migration and invasion assays

Cell migration and invasion experiments were conducted by transwell chambers with 8 mm pores (Corning, Cambridge, MA) according to previous description.^6^

### 2.8 Statistical analysis

SPSS statistics 17.0 software (SPSS Inc, Chicago, IL) was used to perform the statistical analysis. The significance of difference between two group quantitative variables was estimated by using Student's *t *tests. The significance of relationship between two categorical variables was evaluated by using Chi‐squared test. Survival analysis was performed through Kaplan‐Meier method and Cox regression models. Spearman's correlation analysis was performed to determine the correlation between MIR503HG expression and miR‐103 expression. A *P* < 0.05 was considered statistically significant.

## 3 RESULTS

### 3.1 MIR503HG is down‐regulated in triple‐negative breast cancer tissues and cell lines

**Figure 1:**
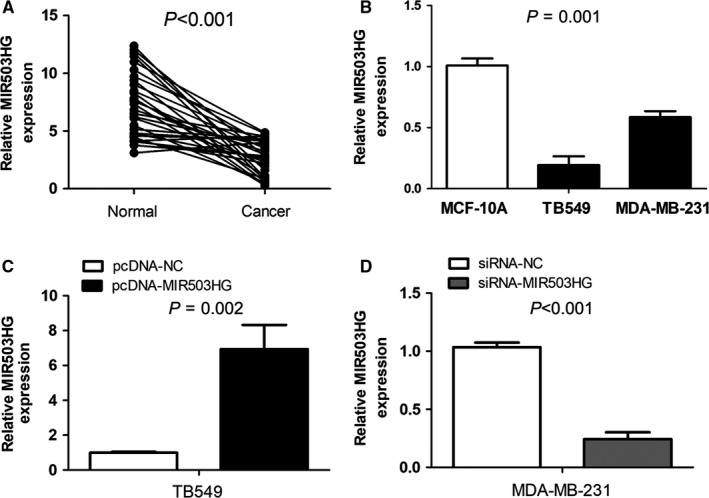
MIR503 host gene (MIR503HG) is down‐regulated in triple‐negative breast cancer tissues and cell lines. (A) MIR503HG expression was markedly decreased in primary triple‐negative breast cancer tissue samples compared with adjacent normal mammary tissue samples. (B) Triple negative breast cancer cell lines exhibited low levels of MIR503HG in contrast to normal breast epithelial cell line. (C) TB549 was transfected with pcDNA‐MIR503HG to overexpress MIR503HG expression, and MDA‐MB‐231 was transfected with siRNA‐MIR503HG to knockdown MIR503HG expression

The relative expression of MIR503HG in primary triple‐negative breast cancer tissue samples and corresponding adjacent normal mammary tissue samples was measured using qRT‐PCR. Compared with adjacent normal mammary tissue samples, MIR503HG expression was markedly decreased in primary triple‐negative breast cancer tissue samples (*P* < 0.001, Figure 1A). Moreover, we also measured the relative expression of MIR503HG in triple negative breast cancer cell lines (MDA‐MB‐231 and TB549) and normal breast epithelial cell line (MCF‐10A), and found triple negative breast cancer cell lines exhibited low levels of MIR503HG in contrast to normal breast epithelial cell line (*P* = 0.001, Figure 1B). To explore the biological effect of MIR503HG on triple negative breast cancer, TB549 was transfected with pcDNA‐MIR503HG for increasing MIR503HG expression, and MDA‐MB‐231 was transfected with siRNA‐MIR503HG for decreasing MIR503HG expression (Figure 1C).

### 3.2 MIR503HG is associated with malignant status of triple‐negative breast cancer patients

For exploring the clinical significance of MIR503HG in triple‐negative breast cancer patient, all cases were classified into the low MIR503HG expression group (n = 47) and the high MIR503HG expression group (n = 47) based on the median expression of MIR503HG.^7^ Correlations between MIR503HG expression and clinicopathological features of triple‐negative breast cancer patients were summarized in Table 1. We observed low MIR503HG expression obviously correlated with clinical stage (I‐II vs III‐IV, *P* < 0.001), lymph node metastasis (N classification, N0‐N1 vs N2‐N3, *P* < 0.001) and distant metastasis (M classification, M0 vs M1, *P* = 0.001). However, MIR503HG expression had no association with age (*P* = 0.365), tumour size (T classification, *P* = 0.055), family history (*P* = 0.135) and histological grade (*P* = 0.835).

**Table 1:** Relationships between MIR503 host gene expression and clinicopathological characteristics in triple‐negative breast cancer

Characteristics	n	Low expression (%)	High expression (%)	*P*
Age(y)				
<50	43	21 (48.8)	22 (51.2)	0.365
≥50	51	16 (39.0)	25 (61.0)	
Clinical stage				
I‐II	35	9 (25.7)	26 (74.3)	<0.001
III‐IV	59	38 (64.4)	21 (35.6)	
T classification				
T1‐T2	59	25 (42.4)	34 (57.6)	0.055
T3‐T4	35	22 (62.9)	13 (37.1)	
N classification				
N0‐N1	43	11 (25.6)	32 (74.4)	<0.001
N2‐N3	51	36 (70.6)	15 (29.4)	
M classification				
M0	81	35 (43.2)	46 (56.8)	0.001
M1	13	12 (92.3)	1 (7.7)	
Family history				
No	81	38 (46.9)	43 (53.1)	0.135
Yes	13	9 (69.2)	4 (30.8)	
Histological grade				
G1	41	21 (51.2)	20 (48.8)	0.835
G2‐G3	53	26 (49.1)	27 (50.9)	

### 3.3 MIR503HG is associated with poor prognosis in triple‐negative breast cancer patients

**Figure 2:**
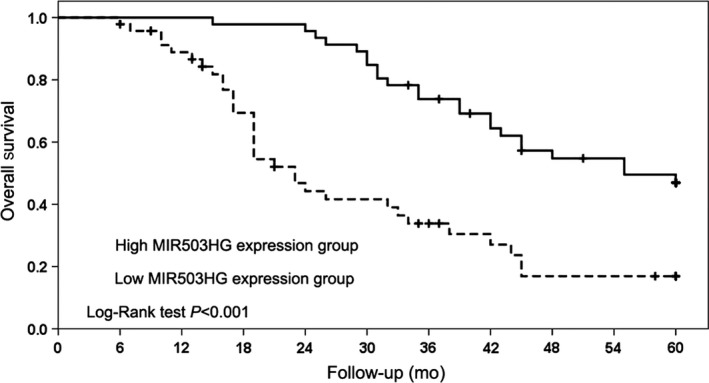
MIR503 host gene (MIR503HG) is associated with poor prognosis in triple‐negative breast cancer patients. The relationship of MIR503HG expression and overall survival was estimated by Kaplan‐Meier method

For exploring the prognostic value of MIR503HG in triple‐negative breast cancer patient, we estimated the relationship of MIR503HG expression and overall survival by Kaplan‐Meier method. We found triple‐negative breast cancer patients with low MIR503HG expression had a statistically significant worse prognosis compared with those with high MIR503HG expression (*P* < 0.001, Figure 2). Furthermore, we evaluated the prognostic factors for overall survival in triple‐negative breast cancer patients, and found clinical stage (*P* = 0.004), T classification (*P* = 0.006), N classification (*P* = 0.016), M classification (*P* < 0.001) and MIR503HG expression (*P* < 0.001) were identified as prognostic factors through univariate Cox regression models (Table 2). Then, low MIR503HG expression was further identified as a poor independent prognostic factor for triple‐negative breast cancer patients through multivariate Cox regression models (*P* = 0.014, Table 2).

**Table 2:** Univariate and multivariate Cox regression analysis for overall survival intriple‐negative breast cancer patients

Parameter	Univariate analysis			Multivariate analysis		
	HR	95% CI	*P*	HR	95% CI	*P*
Age (<50 vs ≥50)	0.821	0.483‐1.395	0.465			
Clinical stage (I‐II vs III‐IV)	2.392	1.318‐4.343	0.004	2.196	0.740‐6.520	0.157
T classification (T1‐T2 vs T3‐T4)	2.117	1.242‐3.610	0.006	1.468	0.828‐2.604	0.188
N classification (N0‐N1 vs N2‐N3)	1.970	1.133‐3.324	0.016	0.480	0.172‐1.340	0.161
M classification (M0 vs M1)	7.793	3.772‐16.100	<0.001	5.311	2.379‐11.853	<0.001
Family history (No vs Yes)	0.791	0.373‐1.678	0.541			
Histological grade (G1 vsG2‐G3)	1.293	0.750‐2.231	0.355			
MIR503HG expression (Low vs High)	0.319	0.185‐0.552	<0.001	0.426	0.216‐0.842	0.014

Abbreviations: HR, hazard ratio; 95% CI, 95% confidence interval.

### 3.4 MIR503HG directly interacts with miR‐103 in triple‐negative breast cancer

**Figure 3:**
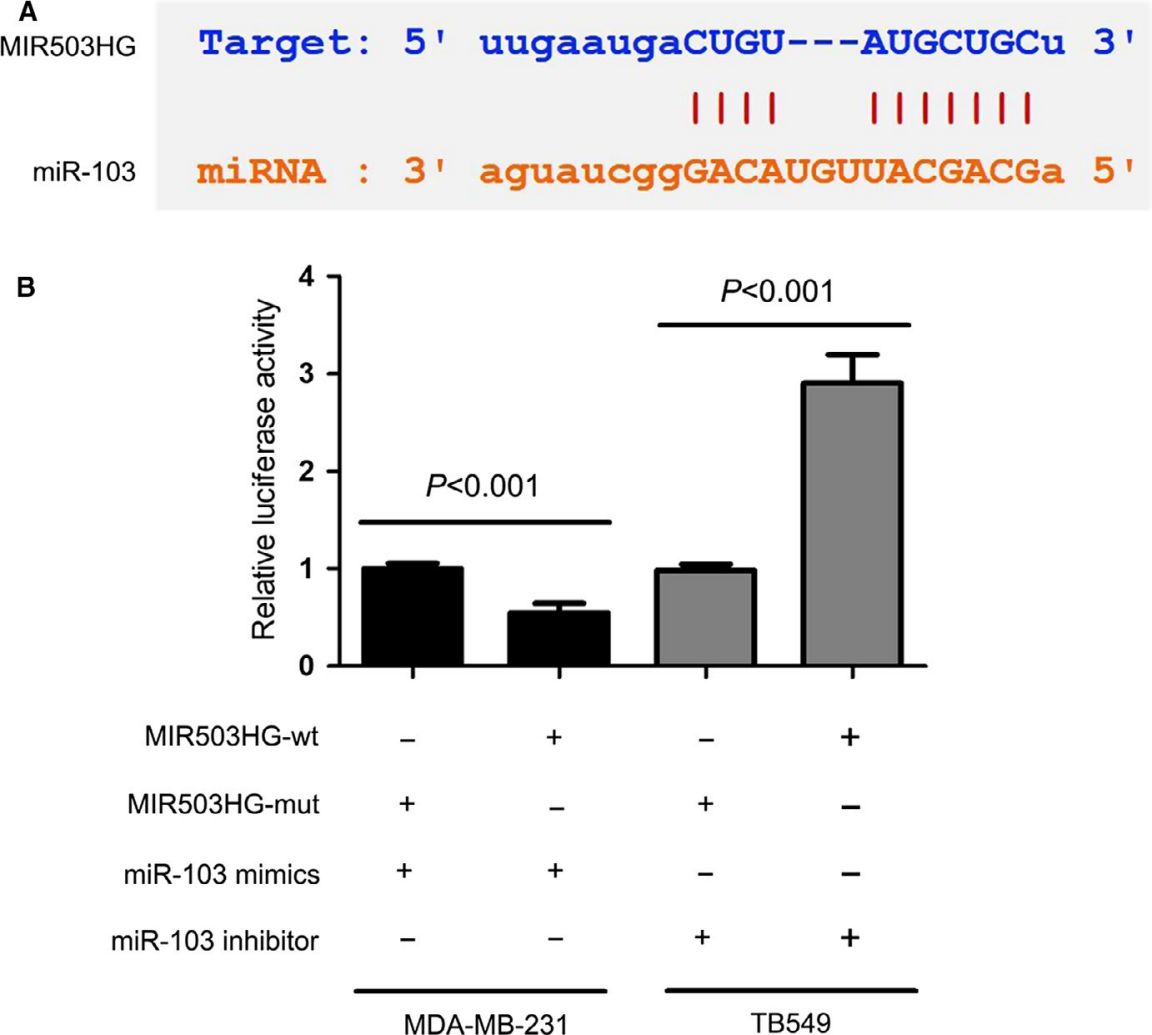
MIR503 host gene (MIR503HG) directly interacts with miR‐103 in triple‐negative breast cancer. (A) Starbase database suggested that there were putative binding sites between MIR503HG and miR‐103. (B) Luciferase reporter gene assay revealed that miR‐103 directly targeted of MIR503HG‐wt to negatively regulate the luciferase activity of MIR503HG‐wt, rather than MIR503HG‐mut

The bioinformatics analysis was conducted using online prediction tool (Starbase, http://starbase.sysu.edu.cn/) to search for lncRNAs paired with miR‐103. The data suggested that there were putative binding sites between MIR503HG and miR‐103 (Figure 3A). Furthermore, luciferase reporter gene assay revealed that miR‐103 directly targeted of MIR503HG‐wt to negatively regulate the luciferase activity of MIR503HG‐wt, rather than MIR503HG‐mut (*P* < 0.001, Figure 3B). The above findings suggested that miR‐103 was a target of MIR503HG in triple‐negative breast cancer cells.

### 3.5 Reciprocal modulation of MIR503HG and miR‐103 in triple‐negative breast cancer

**Figure 4:**
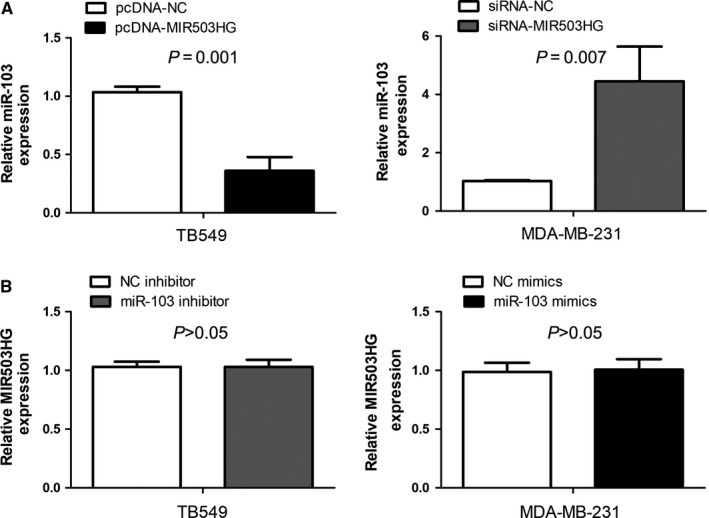
Reciprocal modulation of MIR503 host gene (MIR503HG) and miR‐103 in triple‐negative breast cancer. (A) The expression of MIR503HG was negatively correlated with miR‐103 expression in triple‐negative breast cancer tissues. (B) MIR503HG negatively regulated miR‐103 expression in triple‐negative breast cancer cells. (C) miR‐103 mimics or miR‐103 inhibitor had no significant effect on MIR503HG expression in triple‐negative breast cancer cells

For further explore the relationship between MIR503HG and miR‐103, the correlation between of them in triple‐negative breast cancer tissues was evaluated by Spearman's correlation analysis. We observed that the expression of MIR503HG was negatively correlated with miR‐103 expression in triple‐negative breast cancer tissues (*P* < 0.001, Table 3). Furthermore, we investigated the effect of MIR503HG on miR‐103 expression in triple‐negative breast cancer cells, and found up‐regulation of MIR503HG expression significantly elevated miR‐103 expression (*P* = 0.001, Figure 4A) and down‐regulation of MIR503HG expression greatly reduced miR‐103 expression (*P* = 0.007, Figure 4A). Moreover, we investigated the impact of miR‐103 on MIR503HG expression in triple‐negative breast cancer cells, and suggested miR‐103 mimics or miR‐103 inhibitor had no significant effect on MIR503HG expression (*P* > 0.05, Figure 4B).

**Table 3:** The association between MIR503 host gene and miR‐103 in triple‐negative breast cancer

Group	MIR503HG		*r*	*P*
	High expression	Low expression		
miR‐103				
High expression	8	39	−0.660	<0.001
Low expression	39	8		

### 3.6 MIR503HG interacts with miR‐103 to regulate cell migration and invasion in triple‐negative breast cancer

**Figure 5:**
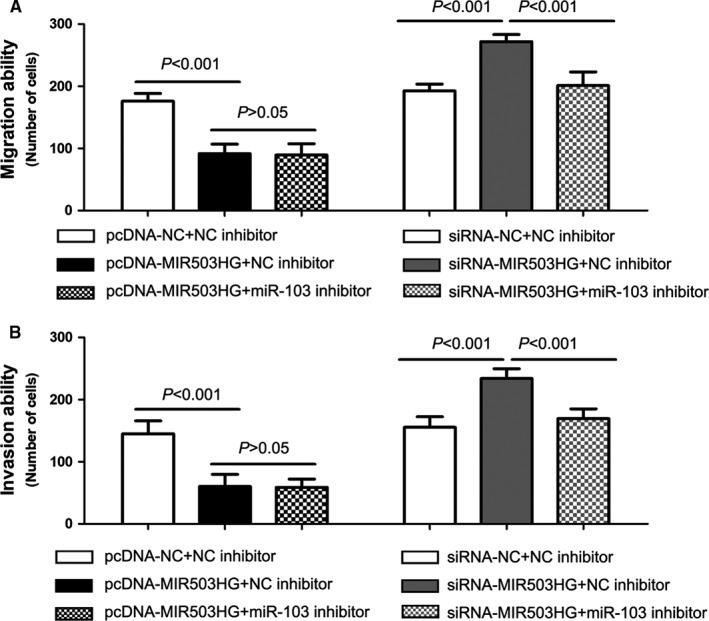
MIR503 host gene (MIR503HG) interacts with miR‐103 to regulate cell migration and invasion in triple‐negative breast cancer. The effects of MIR503HG and miR‐103 on triple‐negative breast cancer cell migration and invasion was evaluated by transwell cell migration (A) and invasion (B) assays

To explore the effects of MIR503HG on triple‐negative breast cancer cell migration and invasion, transwell cell migration and invasion assays were conducted. The results showed up‐regulation of MIR503HG expression led to a decrease of cell migration and invasion ability in TB549 cell, and down‐regulation of MIR503HG expression led to a increase of cell migration and invasion ability in MDA‐MB‐231 cell (*P* < 0.001, Figure 5A,B). Moreover, rescue experiments were conducted to interpret whether MIR503HG regulated cell migration and invasion via miR‐103 in triple‐negative breast cancer. We found co‐transfection of siRNA‐MIR503HG and miR‐103 inhibitor could rescue the facilitation of siRNA‐MIR503HG in cell migration and invasion (*P* < 0.001, Figure 5A,B), and co‐transfection of pcDNA‐MIR503HG and miR‐103 inhibitor could not enhance the inhibition of pcDNA‐MIR503HG in cell migration and invasion (*P* > 0.05, Figure 5A,B).

### 3.7 OLFM4 as the target gene of miR‐103 is modulated by MIR503HG in triple‐negative breast cancer

**Figure 6:**
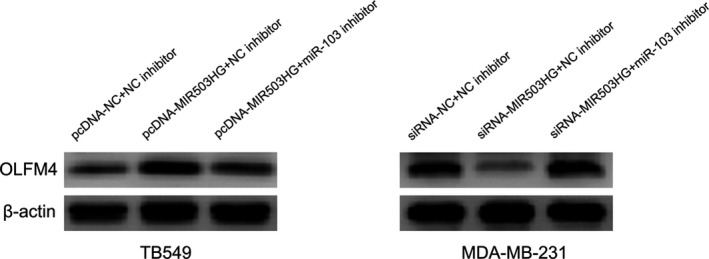
Olfactomedin 4 (OLFM4) as the target gene of miR‐103 is modulated by MIR503 host gene (MIR503HG) in triple‐negative breast cancer. The effects of MIR503HG and miR‐103 on OLFM4 expression was assessed by Western blot in triple‐negative breast cancer cells

Our previous report suggested miR‐103 functioned as oncogenic microRNA to modulate triple negative breast cancer cell migration and invasion through targeting OLFM4 expression.^7^ Above results showed MIR503HG directly negatively regulated miR‐103 in triple‐negative breast cancer. Then, we attempted to explore whether MIR503HG regulates cell migration and invasion by miR‐103/OLFM4 axis. Western blot suggested pcDNA‐MIR503HG obviously increased OLFM4 expression in TB549 cell, and miR‐103 inhibitor could not further enhance the facilitation of pcDNA‐MIR503HG on OLFM4 expression. Meanwhile, siRNA‐MIR503HG notably decreased OLFM4 expression in MDA‐MB‐231 cell (Figure 6), and miR‐103 inhibitor could rescue the inhibition of siRNA‐MIR503HG on OLFM4 expression (Figure 6).

## 4 DISCUSSION

MIR503HG is the host gene of miR503, and located on chromosome Xq26.3, which is a region enriched for genes associated with human reproduction.^11^ Originally, MIR503HG was identified to be involved in human endothelial cells and angiogenic processes.^12^ Soon afterwards, Muys et al^13^ found MIR503HG expression was increased in placenta, other reproductive tissues and 50% cancer cell lines. Then, Zhang et al^14^ found MIR503HG as an up‐regulated lncRNA in colorectal cancer tissues through analysing RNA‐seq datasets. Moreover, Chung et al^15^ performed oligonucleotide arrays for identifying aberrantly expressed lncRNAs, and found MIR503HG was one of top‐five non‐coding genes, and showed high expression levels in anaplastic large‐cell lymphoma. On the contrary, MIR503HG was suggested to be down‐regulated in hepatocellular carcinoma 16 and oral squamous cell carcinoma.^17^ However, the MIR503HG expression in breast cancer was still unknown. In our study, we conducted qRT‐PCR to measured the relative expression of MIR503HG in triple‐negative breast cancer tissue samples and cell lines, and found MIR503HG expression was significantly decreased in triple‐negative breast cancer tissues and lines compared with adjacent normal mammary tissue samples and normal breast epithelial cell line, respectively. Furthermore, we investigated the clinical value of MIR503HG in triple‐negative breast cancer patient by analysing correlations between MIR503HG expression and clinicopathological features, and found low MIR503HG expression was correlated with late clinical stage, lymph node metastasis and distant metastasis. In the survival analysis, we observed that triple‐negative breast cancer patients with low MIR503HG expression had a statistically significant worse prognosis compared with those with high MIR503HG expression, and low MIR503HG expression was a poor independent prognostic factor for overall survival in triple‐negative breast cancer patients. Similar, Wang et al^16^ reported low‐expression of MIR503HG was markedly correlated with poor time to recurrence (TTR) and overall survival and served as an independent unfavourable risk factor for TTR and overall survival in hepatocellular carcinoma patients. In addition, Alaei et al^18^ suggested MIR503HG was selected as candidate lncRNAs for predicting overall survival in oesophageal squamous‐cell carcinoma, but the detailed results are not shown in the article.

In our previous study, we identified OLFM4 as tumour‐suppressing gene to be correlated with present lymph node metastasis, distant metastasis, advanced clinical stage and poor prognosis and suppress cell migration and invasion in triple negative breast cancer.^6^ Meanwhile, we found miR‐103 functioned as oncogenic microRNA to modulate triple negative breast cancer cell migration and invasion through targeting OLFM4 expression.^7^ Interestingly, we found MIR503HG has potential binding sites for miR‐103 at online prediction tool (Starbase v2.0), and MIR503HG expression had a negative correlation with miR‐103 expression in triple negative breast cancer. Thus, we had a hypothesis that MIR503HG inhibits cell migration and invasion via miR‐103/OLFM4 axis in triple negative breast cancer. First, we confirmed that MIR503H directly interacts with miR‐103 and negatively regulated miR‐103 expression, but miR‐103 expression had no significant effect on MIR503HG expression in triple negative breast cancer cells. Second, we conducted loss‐ and gain‐of‐function experiments, and found MIR503HG expression inhibited cell migration and invasion in triple negative breast cancer cells. Third, we performed rescue experiments, and showed MIR503HG interacts with miR‐103 to regulate cell migration and invasion in triple‐negative breast cancer. Finally, we executed Western blot for exploring the effect of MIR503HG/miR‐103 on OLFM4 expression, and found OLFM4 as the target gene of miR‐103 was also modulated by MIR503HG in triple‐negative breast cancer. Based on above results, we thought that MIR503HG inhibits cell migration and invasion via miR‐103/OLFM4 axis in triple negative breast cancer. In addition, Wang et al^16^ indicated MIR503HG suppressed hepatocellular carcinoma cell invasion and metastasis in vitro and in vivo through modulating HNRNPA2B1/NF‐κB pathway. Moreover, Muys et al^13^ showed up‐regulation of MIR503HG inhibited cell migration and invasion of JEG‐3 choriocarcinoma cells. However, Huang et al^19^ revealed that MIR503HG promoted tumour cell proliferation and growth in vitro and in vivo through regulating miR‐503/Smurf2/TGFBR axis in anaplastic large‐cell lymphoma.

In conclusion, Low MIR503HG expression is observed in triple‐negative breast cancer tissues and cells, and associated with malignant status and unfavourable overall survival in triple‐negative breast cancer patients. MIR503HG inhibits cell migration and invasion via miR‐103/OLFM4 axis in triple negative breast cancer.

## CONFLICT OF INTEREST

The authors declare no conflict of interest.

## AUTHOR CONTRIBUTION

Jia Fu and Bin Xiong: designed the experiment, interpreted the data, and prepared the manuscript. Jia Fu, Guanjun Dong, Hui Shi, Junfeng Zhang, Zhaochen Ning, Xingna Bao, Chenjie Liu, Jing Hu and Minghui Liu: conducted the experiment, collected the data, and reviewed the manuscript.
